# Psychological well-being of rural left-behind women in Northwest China and its associated factors: a regional, population-based study

**DOI:** 10.3389/fpubh.2024.1395996

**Published:** 2024-12-19

**Authors:** Fang Niu, Xiang Wang

**Affiliations:** ^1^School of Philosophy and Sociology, Lanzhou University, Lanzhou, China; ^2^Medical Psychological Center, The Second Xiangya Hospital of Central South University, Changsha, China; ^3^Faculty of Medicine and Health Sciences, Department of Psychiatry, McGill University, Montreal, QC, Canada

**Keywords:** rural left-behind women, depression, anxiety, feeling of security, associated factors

## Abstract

**Purpose:**

Growing awareness has highlighted the challenging living condition faced by rural left-behind women (RLW), yet their psychological well-being has not been fully investigated. This study aims to investigate the psychological well-being of RLW in Northwest China and exploring its associated factors.

**Samples and methods:**

A total of 697 RLW from five provincial regions were enrolled. Sociodemographic characteristics were collected using a set of researcher-designed questionnaires. Depression, anxiety, and feeling of security were assessed using the Zung’s self-rating depression scale (SDS), Zung’s self-rating anxiety scale (SAS), and security questionnaire (SQ), respectively.

**Results:**

The prevalences of depression and anxiety among RLW were 35.7 and 37.6%, respectively, and feelings of security was relatively low in RLW, with a mean SQ score of 50.16 ± 11.37. Chi-square tests and multiple linear regression analyses indicated that labor intensity, physical health conditions, marital satisfaction and stability, relationships with children, frequency of husband coming home, left-behind duration, domestic violence, and sexual harassment after husbands left were risk factors of psychological well-being of RLW.

**Conclusion:**

These findings revealed that the psychological well-being of RLW in Northwest China is not promising, which should therefore, be given special attention. It is essential to prioritize the improvement of the psychological well-being for RLW by providing accessible and targeted supports and interventions tailored to cope with their challenges.

## Introduction

With the reform and development of China’s society and economy, a substantial influx of male rural laborers has occurred as cities strive to meet the demands of rapid development. However, due to economic constraints faced by migrant workers, the option of “family relocation” remains unattainable. Consequently, male laborers from rural areas migrate to cities for employment while their family members were left in rural areas thus forming a special rural left-behind population, including the older adult, women, and children, in the Chinese society. Among them, rural left-behind women (RLW) play a vital role in rural areas. Due to their husbands’ absence, the RLW must take on multiple responsibilities, such as agricultural production, taking care of the children and seniors, and running family chores at home (1), thus contributing significantly to daily production activities and ensuring the smooth functioning of rural society.

The term of RLW in China specifically denotes rural women who remain at home while their husbands migrate for work, business, or other productive activities typically for prolonged durations (at least 6 months) (2). The number of RLW in China is estimated to exceed 50 million (3), who have to deal with livelihood fragility (4). Extensive research has consistently revealed that RLW in China experience severe challenges in terms of their overall well-being and survival (1–3, 5, 6). The most striking feature of RLW in China is that they bear what is often referred to as the “three big mountains,” that is high level of mental pressure, high feeling of insecurity, and heavy burden of labor (7). Due to the absence of their husbands, RLW carry significant burdens as they shoulder the responsibility of caring for both left-behind older adult and left-behind children, in addition to managing demanding tasks such as agricultural production and household chores. Left-behind women are not unique to China. Indeed, a substantial body of research on this topic has been made in underdeveloped countries, including regions such as South America, South and Southeast Asia, and Africa. In foreign countries, the focus tends to be on the broader category of “left-behind women,” without making a specific distinction between urban and rural areas, and these studies primarily concentrated on their survival status, personal safety, gender identity, and psychological and social changes they undergo following the departure of their husbands (8–11). There is no doubt that the multiple responsibilities and challenges borne by left-behind women accompanied by the departure of their husbands inevitably caused great psychological pressure on them. Consequently, they are more vulnerable to psychological problems, such as negative moods, insecurity, as well as mental disorders (1, 5–7, 11). Hence, it is crucial to prioritize attention toward the psychological well-being of RLW.

In recent years, while there has been an increasing focus on the psychological well-being of RLW in China, it has not been fully investigated. Some previous studies have been qualitative in nature (7), and some studies only described specific negative moods (e.g., depression) (1, 6, 12) or psychological variables (e.g., copy strategy, resilience) accompanied by other issues (2, 13), rather than provide an overall picture of their psychological well-being and exploring the potential risk or protective factors. However, RLW in the remote areas of Northwest China have received insufficient attention so far. The remote geographical location and underdeveloped economy of Northwest China have resulted in higher rates of population mobility compared to other regions, thereby producing a larger population base of RLW. Meanwhile, the remote location of the areas in Northwest China results in the fact that the husbands often working far away from home, making it more difficult for them to return home frequently and assist their wives in sharing the responsibilities and pressures that they encounter. Thus, the living conditions of RLW in Northwest China may be more challenging and severe. Moreover, Northwest China is characterized by a diverse range of ethnic groups. There are great differences among ethnic groups in terms of ethnic culture and living habits, and many ethnic minorities in this region hold religious beliefs, which is a significant difference from other regions. Previous studies have shown that religious beliefs could improve the ability of one to cope with stress and hardship, depression, and anxiety (14), it is unclear whether the religious beliefs could be a protective factor of psychological well-being in RLW. Therefore, it is of great significance to prioritize attention toward the psychological well-being of RLW in Northwest China, considering the distinct challenges they face in this particular context.

Given the lack of a large-scale population-based study on the psychological well-being of RLW in China and the special situation in the Northwest region, the current study aims to investigate the psychological well-being of RLW in Northwest China and explore its associated factors. The present study was expected to provide valuable insights for government departments and relevant organizations to implement measures focused on improving the psychological well-being and overall living conditions of RLW by investigating the conditions of psychological well-being and exploring the potential associated factors among RWL in Northwest China.

## Methods

### Participants and survey

Participants in the present study were recruited in five provincial administrative regions of Northwest China (Shanxi, Gansu, Ningxia, Qinghai, and Xinjiang) by using simple random sampling, convenient sampling, discriminant sampling, and snowball technique. The administrative village was taken as sampling units, we first randomly selected two cities/counties from each provincial administrative region; then, a township from each city/county was selected; finally, a village was selected from each township. Considering the particularity of Xinjiang, we selected 2 villages from one city in Xinjiang. Therefore, 10 villages were selected as study sites (see Table 1 for details).

**Table 1:** Distribution of study sites.

No.	Province	City/County	Township	Village	Valid sample
1	Gansu	Linxia / DX	HT	WH	72
2	Gansu	Longxi / LX	GC	YH	70
3	Shanxi	Baoji / CC	DG	DGQ	88
4	Shanxi	Shangluo /SZ	SHZ	WS	83
5	Ningxia	Yinchuan / XQ	YYH	BHJY	72
6	Ningxia	Guyuan / PY	MY	SS	63
7	Qinghai	Xining / HZ	LSE	HYG	67
8	Qinghai	Haidong / HZ	NMX	XK	83
9	Xinjiang	Yili / XY	KLBL	AKTG	70
10	Xinjiang	Yili / XY	KLBL	KMAWZ	29

We distributed survey questionnaires by convenient sampling first through door-to-door visits. We conducted simple communications with local women to determine whether they were suitable for the survey before formally performing the survey. According to the definition of RLW in China (2), women whose husbands have migrate to urban areas for work at least six months to a year at a time were enrolled in our survey. We further investigate more RLW in a snowball-like manner based on the information provided by RLW who had been surveyed. We excluded the subjects who had difficulties in communicating. We provided survey information on the questionnaires, all participants agreed to participate in the survey and the written informed consents were provided before completing the measures. One-by-one interviews with the selected women were conducted by investigators, and the investigation and recycling work were completed on-site. Participants who completed the survey were given compensation by cash or its equivalent. A total of 711 RLW were recruited and 697 of them fully completed our survey, with an effective response rate is 98.03% (see Table 1 for details).

### Measure

#### General data questionnaire

Information on age, ethnic group, religious belief, education level, marital status (duration, stability, etc.), physical health, duration of left-behind, frequency of husband coming home, the number of children, pressure from children’s education, relationship with children, relationship with parents-in-law, labor intensity, domestic violence, and sexual harassment after husbands left were collected.

#### Psychological well-being

Depression and anxiety were measured using Zung’s self-rating depression scale (SDS) (15) and Zung’s self-rating anxiety scale (SAS) (16). Both SDS and SAS contain 20 self-rating items, using a 4-point Likert scale ranging from 1 (none, or a little of the time) to 4 (most, or all of the time) to assess depression and anxiety level within the past week. Index scores of SDS and SAS could be calculated by multiplying the raw score by 1.25, ranging from 25 to 100. Higher SDS and SAS scores indicate more severe depression and anxiety levels. The cut-off score of SAS is index score ≥ 50; 50–59, 60–69, and ≥ 70 indicate mild, moderate, and severe anxiety, respectively. Depression severity is assessed by SDS indices (raw score / 80), ranging from 0.25–1.0. The cut-off score of SDS indices is 0.5; 0.50–0.59, 0.6–0.69, and ≥ 0.7 indicate mild, moderate, and severe depression, respectively. In the current study, SAS score ≥ 50 and SDS indices ≥0.5 were used as cut-off scores for assessing the prevalence of anxiety and depression. Cronbach’s *α*(s) for the current sample were 0.845 and 0.867 for SDS and SAS, respectively.

Feeling of security was measured using Security questionnaire (SQ), which was developed by Cong and An (17). SQ contains 16 self-reporting items, using a 5-point Likert scale ranging from 1 (strongly agree) to 5 (strongly disagree), and can yield two factors that evaluate two aspects of feeling of security: interpersonal security (IS) and certainty in control (CC). A Higher score indicates a stronger feeling of security. Cronbach’s *α*(s) for the current sample was 0.777 for SQ.

### Statistical analysis

SPSS 29.0 was used to conduct statistical analysis. Since the missing rates of all variables were less than 5%, we used the mean value (for continuous variables) or median value (for ordinal variables) for imputation. Harman’s one-factor test for common-method variance bias was performed given that psychological well-being indexes were collected by using self-rated questionnaires (18). One-way ANOVAs were used for testing the differences in psychological well-being among RLW from different provinces and different ethnic groups. In order to build more accurate predictive models, we first used Chi-square tests to identify potential predictors of depression and anxiety; and used Pearson correlation analysis to identify potential predictors of feeling of security. Then, all these potential predictors were put into multiple linear regression models to explore the associated factors of psychological well-being among RLW. A *p* < 0.05 was considered statistically significant.

## Results

Descriptive statistics of all observed variables are presented in Table 2. The mean scores of SDS and SAS of RLW are 45.41 ± 12.49 and 46.27 ± 12.89, respectively. We further explored the depression and anxiety status of RLW and showed that 35.7% (*n* = 249) of them reported depression status, and 37.6% (*n* = 262) of them reported anxiety status. For depression severity, 18.9% (*n* = 132) of RLW showed mild depression, 13.2% (*n* = 92) of them showed moderate depression, 3.6% (*n* = 25) of them showed severe depression. For anxiety severity, 22.4% (*n* = 156) of RLW showed mild anxiety, 9.6% (*n* = 67) of them showed moderate anxiety, 5.6% (*n* = 39) of them showed severe depression. The mean scores of the SQ, IS subscale, and CC subscale are 50.16 ± 11.37, 25.15 ± 6.05, and 25.00 ± 6.94, respectively. Result of Harman’s one-factor test indicated that the eigenvalues of 13 factors in the present study are greater than 1, and the first factor can only explain 21.109%, which is far less than the accumulated explanatory variance of 40%. Therefore, no serious common method bias problem exists in this study.

**Table 2:** Characteristics of the participants (*n* = 697).

Characteristics	*N*	%	Characteristics	N	%
**Age**			**Ethnic group**		
≤ 25 years old	49	7.0	Han	424	60.8
26–35 years old	200	28.7	Hui	112	16.1
36–45 years old	222	31.9	Uighur	10	1.4
46–55 years old	175	25.1	Tibetan	58	8.3
≥ 56 years old	51	7.3	Other	93	13.3
**Education level**			**Physical health**		
Illiteracy	222	31.9	Completely health	187	26.8
Primary school	212	30.4	General health	178	25.5
Middle school	224	32.1	Have common diseases	263	37.7
High school or above	39	5.6	Have serious diseases	69	9.9
**Religious beliefs**			**The number of children**		
Yes	338	48.5	No child	15	2.2
No	359	51.5	1 child	143	20.5
**Relationships with children**			2 children	372	53.4
Good	673	96.6	3 children	125	17.9
Fair or bad	24	3.4	4 or more children	42	6.0
**Education pressure of children**			**Frequency of husband coming home**		
High pressure	341	48.9	Very frequently	164	23.5
Relatively high pressure	109	15.6	Relatively frequently	104	14.9
Average pressure	65	9.3	Average	214	30.7
Relatively low pressure	32	4.6	Relatively infrequently	144	20.7
Low pressure	93	13.3	Very infrequently	71	10.2
No child in school ^a^	57	8.2			
**Marriage duration**			**Left-behind duration**		
≤ 5 years	76	10.9	≤ 5 years	229	33.2
6–10 years	118	16.9	6–10 years	153	20.8
11–15 years	103	14.8	11–15 years	97	13.9
16–20 years	100	14.3	16–20 years	52	7.5
≥ 20 years	300	43.0	≥ 20 years	166	23.8
**Marital satisfaction**			**Marital stability**		
Very or somewhat satisfied	580	83.2	Very or relatively stable	634	91.0
Average	92	13.2	Average	45	6.4
Very or somewhat dissatisfied	25	3.6	Very or relatively unstable	18	2.6
**Relationships with parents-in-law**			**Labor intensity**		
Good	555	79.6	High	377	54.1
Fair or bad	142	20.4	Moderate	174	25.0
			Low	146	20.9
**Domestic violence**			**Sexual harassment**		
Yes	233	33.4	Yes	39	5.6
No	464	66.6	No	658	94.4

^aFifty-seven participants reported they have no children currently in school, thus rating for education pressure wasn’t applicable.^

**Figure 1:**
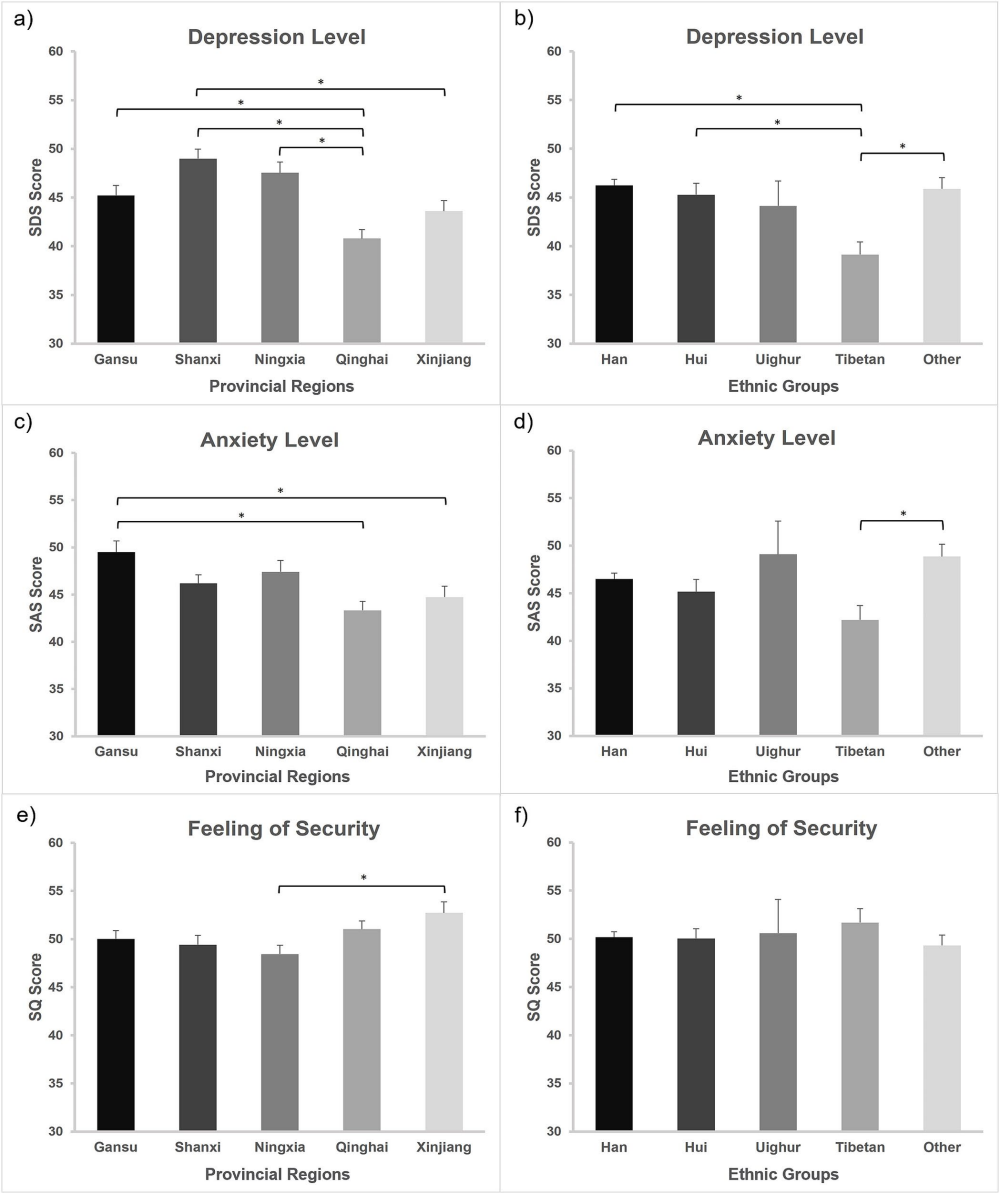
Psychological well-beings among different provincial regions and ethnic groups. The levels of **(A)** depression, **(C)** anxiety, and **(E)** feeling of security of rural left-behind women among different regions; the levels of **(B)** depression, **(D)** anxiety, and **(F)** feeling of security of rural left-behind women among different ethnic groups. Data are presented as the mean ± s.e. *p < 0.05.

For the differences among different provincial regions in psychological well-being, RLW in Gansu, Shanxi, and Ningxia showed higher depression levels than those who come from Qinghai, and RLW in Shanxi have higher depression levels than those in Xinjiang; RLW in Gansu have higher anxiety levels than those in Qinghai and Xinjiang; RLW in Xinjiang showed higher feelings of security than those in Ningxia. For the differences in psychological well-being among different ethnic groups, RLW from Han, Hui, and other ethnic groups showed higher depression levels than Tibetan RLW; and RLW from other ethnic group have higher anxiety levels than Tibetan RLW. We did not find differences in feelings of security among different ethnic groups (see Figure 1 and Supplementary Table S1). It is noted that comparisons across provinces and ethnic groups consistently revealed the lowest depression and anxiety level of RLW in Qinghai or RLW who were Tibetan, and Qinghai is the Tibetan settlement area. In the current study, half of the RLW from Qinghai province are Tibetan Buddhist believers. These findings raised our interests in regard to why Tibetan showed more positive results in psychological well-being than other ethnic groups. We therefore explored whether the psychological well-being indexes are associated with specific religious belief, such as Tibetan Buddhism, Islam, and other religious beliefs (see Supplementary Table S2). The results showed that RLW with Tibetan Buddhism belief exhibited significantly lower depression level than RLW with Islam and other religious beliefs, and marginal significantly lower than RLW with non-religious beliefs (*p* = 0.076). Meanwhile, RLW with Tibetan Buddhism belief also showed highest feeling in security, although the differences were not significant. No significant differences in anxiety level were found across groups with different religious beliefs.

To determine the potential associated factors of depression and anxiety among RLW, Chi-square tests were used. As shown in Table 3, the prevalence of depressive symptoms and anxiety symptoms in RLW showed difference among different age groups, physical health status, education pressure of children, marriage duration, marital satisfaction, marital stability, frequency of husband coming home, labor intensity, relationship with parents-in-law, and domestic violence. In addition, the prevalence of depressive symptoms was also associated with the relationships with children and sexual harassment after husbands left; and the prevalence of anxiety symptoms was associated with education level, and the number of children. Therefore, these variables were included in subsequent multiple linear regression models to further explore the associated factors of depression and anxiety among RLW.

**Table 3:** Effects of different variables on depression and anxiety status (*n* = 697).

Variables	Non-DEP (%)	DEP (%)	*χ^2^*	*p*	Non-ANX (%)	ANX (%)	*χ^2^*	*p*
**Age**			**20.819**	**< 0.001**			**33.477**	**< 0.001**
≤ 25 years old	35 (71.4)	14 (28.6)			39 (79.6)	10 (20.4)		
26–35 years old	143 (71.5)	57 (28.5)			143 (71.5)	57 (28.5)		
36–45 years old	150 (67.6)	72 (32.4)			135 (60.8)	87 (39.2)		
46–55 years old	97 (55.4)	78 (44.6)			101 (57.7)	74 (42.3)		
≥ 56 years old	23 (45.1)	28 (54.9)			17 (33.3)	34 (66.7)		
**Education level**			3.702	0.296			**7.82**	**0.050**
Illiteracy	142 (64.0)	80 (36.0)			122 (55.0)	100 (45.0)		
Primary school	146 (68.9)	66 (31.1)			138 (65.1)	74 (34.9)		
Middle school	138 (61.6)	86 (38.4)			149 (66.5)	75 (33.5)		
High school or above	22 (56.4)	17 (43.6)			26 (66.7)	13 (33.3)		
**Religious beliefs**			0.002	0.968			1.546	0.214
Yes	217 (64.2)	121 (35.8)			203 (60.1)	135 (39.9)		
No	231 (64.3)	128 (35.7)			232 (64.6)	127 (35.4)		
**Physical health**			**45.014**	**< 0.001**			**96.562**	**< 0.001**
Completely health	150 (80.2)	37 (19.8)			156 (83.4)	31 (16.6)		
General health	119 (66.9)	59 (33.1)			128 (71.9)	50 (28.1)		
Have common diseases	152 (57.8)	111 (42.2)			133 (50.6)	130 (49.4)		
Have serious diseases	27 (39.1)	42 (60.9)			18 (26.1)	51 (73.9)		
**The number of children**			6.007	0.199			**25.867**	**< 0.001**
No child	8 (53.3)	7 (46.7)			8 (53.3)	7 (46.7)		
1 child	98 (68.5)	45 (31.5)			109 (76.2)	34 (23.8)		
2 children	243 (65.3)	129 (34.7)			236 (63.4)	136 (36.6)		
3 children	78 (62.4)	47 (37.6)			64 (51.2)	61 (48.8)		
4 or more children	21 (50.0)	21 (50.0)			18 (42.9)	24 (57.1)		
**Education pressure of children** ^**a**^			**16.445**	**0.002**			**9.492**	**0.050**
High pressure	215 (63.0)	126 (37.0)			200 (58.7)	141 (41.3)		
Relatively high pressure	64 (58.7)	45 (41.3)			65 (59.6)	44 (40.4)		
Average pressure	35 (53.8)	30 (46.2)			41 (63.1)	24 (36.9)		
Relatively low pressure	23 (71.90)	9 (28.1)			22 (68.8)	10 (31.3)		
Low pressure	75 (80.6)	18 (19.4)			70 (75.3)	23 (24.7)		
**Marriage duration**			**15.176**	**0.004**			**32.473**	**< 0.001**
≤ 5 years	54 (71.1)	22 (28.9)			58 (76.3)	18 (23.7)		
6–10 years	89 (75.4)	29 (24.6)			90 (76.3)	28 (23.7)		
11–15 years	69 (67.0)	34 (33.0)			67 (65.0)	36 (35.0)		
16–20 years	65 (65.0)	35 (35.0)			66 (66.0)	34 (34.0)		
≥ 20 years	171 (57.0)	129 (43.0)			154 (51.3)	146 (48.7)		
**Marital satisfaction**			**29.868**	**< 0.001**			**12.266**	**0.002**
Very or somewhat satisfied	396 (68.3)	184 (31.7)			378 (65.2)	202 (34.8)		
Average	46 (50.0)	46 (50.0)			47 (51.1)	45 (48.9)		
Very or somewhat dissatisfied	6 (24.0)	19 (76.0)			10 (40.0)	15 (60.0)		
**Marital stability**			**21.116**	**< 0.001**			**11.457**	**0.003**
Very or relatively stable	424 (66.9)	210 (33.1)			408 (64.4)	226 (35.6)		
Average	16 (35.6)	29 (64.4)			20 (44.4)	25 (55.6)		
Very or relatively unstable	8 (44.4)	10 (55.6)			7 (38.9)	11 (61.1)		
**Left-behind duration**			2.399	0.663			6.445	0.168
≤ 5 years	139 (60.7)	90 (39.3)			137 (59.8)	92 (40.2)		
6–10 years	100 (65.4)	53 (34.6)			104 (68.0)	49 (32.0)		
11–15 years	67 (69.1)	30 (30.9)			65 (67.0)	32 (33.0)		
16–20 years	34 (65.4)	18 (34.6)			35 (67.3)	17 (32.7)		
≥ 20 years	108 (65.1)	58 (34.9)			94 (56.6)	72 (43.4)		
**Frequency of husband** **coming home**			**13.390**	**0.010**			**9.776**	**0.044**
Very frequently	114 (69.5)	50 (30.5)			112 (68.3)	52 (31.7)		
Relatively frequently	63 (60.6)	41 (39.4)			62 (59.6)	42 (40.4)		
Average	140 (65.4)	74 (34.6)			141 (65.9)	73 (34.1)		
Relatively infrequently	98 (68.1)	46 (31.9)			85 (59.0)	59 (41.0)		
Very infrequently	33 (46.5)	38 (53.5)			35 (49.3)	36 (50.7)		
**Labor intensity**			**52.060**	**< 0.001**			**85.791**	**< 0.001**
High	198 (52.5)	179 (47.5)			177 (46.9)	200 (53.1)		
Moderate	129 (74.1)	45 (25.9)			134 (77.0)	40 (23.0)		
Low	121 (82.9)	25 (17.1)			124 (84.9)	22 (15.1)		
**Relationships with parents-in-law**			**8.982**	**0.003**			**6.996**	**0.008**
Good	372 (67.0)	183 (33.0)			360 (64.9)	195 (35.1)		
Fair or bad	76 (53.5)	66 (46.5)			75 (52.8)	67 (47.2)		
**Relationships with children**			**7.761**	**0.005**			1.632	0.201
Good	439 (65.2)	234 (34.8)			423 (62.9)	250 (37.1)		
Fair or bad	9 (37.5)	15 (62.5)			12 (50.0)	12 (50.0)		
**Domestic violence**			**4.573**	**0.032**			**15.502**	**< 0.001**
Yes	137 (58.8)	96 (41.2)			122 (52.4)	111 (47.6)		
No	311 (67.0)	153 (33.0)			312 (67.7)	149 (32.3)		
**Sexual harassment**			**7.698**	**0.006**			2.181	0.140
Yes	17 (43.6)	22 (56.4)			20 (51.3)	19 (48.7)		
No	431 (65.5)	227 (34.5)			415 (63.1)	243 (36.9)		

^aFifty-seven participants reported they have no children currently in school, thus only 640 participants were include in chi-square test.^

DEP, depression status; ANX, anxiety status. Bold values indicate statistically significant.

To determine the potential influencing factors of the feeling of security among RLW, Pearson correlation analysis was used and indicated that left-behind duration (*r* = 0.087, *p* < 0.05), marital satisfaction (*r* = −0.166, *p* < 0.001), marital stability (*r* = −0.149, *p* < 0.001), the number of children (*r* = −0.078, *p* < 0.05), education pressure of children (*r* = 0.091, *p* < 0.05), physical health status (*r* = −0.254, *p* < 0.001), labor intensity (*r* = 0.311, *p* < 0.001), relationships with parents-in-law (*r* = −0.132, *p* < 0.001), relationships with children (*r* = −0.103, *p* < 0.01), domestic violence (*r* = 0.125, *p* < 0.001), as well as sexual harassment after husbands left (*r* = 0.136, *p* < 0.001) were associated with the feeling of security. These variables were included in subsequent multiple linear regression models to further explore the associated factors of feeling of security among RLW.

The multiple linear regression model for each psychological well-being outcome was constructed using the stepwise method. As shown in Table 4, M1 explained 26.4% of the variation in SDS scores, and five variables were significantly associated with SDS scores (labor intensity, physical health, marital satisfaction, relationships with children, and sexual harassment after husbands left). M2 explained 33.2% of the variation in SAS scores, and five variables were significantly associated with SAS scores (physical health, labor intensity, marital stability, frequency of husband coming home, and domestic violence). M3 explained 18.9% of the variation in SQ scores, and seven variables were significantly associated with SQ scores (labor intensity, physical health, sexual harassment after husbands left, marital stability, left-behind duration, relationships with children, and domestic violence).

**Table 4:** Multiple liner regression models of SDS, SAS, and SQ (*n* = 697).

	Variable statistics				Model statistics		
Predictors	*B* (*s.e.*)	*β*	*t*	*p*	*R^2^*	*F*	*p*
**SDS (M1)**					0.264	49.479	< 0.001
Labor intensity	−4.728 (0.524)	−0.303	−9.021	< 0.001			
Physical health	3.113 (0.434)	0.243	7.172	< 0.001			
Marital satisfaction	4.863 (0.857)	0.188	5.675	< 0.001			
Relationships with children	6.111 (2.240)	0.089	2.728	0.007			
Sexual harassment	−2.193 (0.893)	−0.081	−2.456	0.014			
**SAS (M2)**					0.332	68.667	< 0.001
Physical health	5.048 (0.430)	0.381	11.743	< 0.001			
Labor intensity	−4.586 (0.516)	−0.285	−8.882	< 0.001			
Domestic violence	−1.449 (0.432)	−0.106	−3.355	< 0.001			
Frequency of husband coming home	0.779 (0.314)	0.078	2.479	0.013			
Marital stability	2.325 (1.029)	0.071	2.259	0.024			
**SQ (M3)**					0.189	22.922	< 0.001
Labor intensity	3.549 (0.503)	0.250	7.063	< 0.001			
Physical health	−2.338 (0.425)	−0.200	−5.505	< 0.001			
Sexual harassment	2.919 (0.857)	0.118	3.406	< 0.001			
Left-behind duration	0.848 (0.255)	0.117	3.323	< 0.001			
Marital stability	−2.991 (1.004)	−0.103	−2.978	0.003			
Relationships with children	−5.698 (2.143)	−0.091	−2.658	0.008			
Domestic violence	0.907 (0.424)	0.075	2.138	0.033			

Only variables that were retained in the model were presented in this table.

SDS, Zung’s self-rating depression scale; SAS, Zung’s self-rating anxiety scale; SQ, security questionnaire.

## Discussion

The present study investigated psychological well-being and explored its associated factors in 697 RLW in Northwest China. The present study found the prevalence of depressive and anxiety symptoms of RLW in Northwest China were 35.7 and 37.6%, respectively. Meanwhile, the average score of the feeling of security in RLW was lower than the general population in China (17). Further analyses found that higher labor intensity and worse physical health conditions were strong risk factors for depression, anxiety, and a lower feeling of security. In addition, depressive symptoms of RLW were also associated with worse marital satisfaction, worse relationships with children, and the existence of sexual harassment after husbands left. Anxiety symptoms of RLW were also related to the existence of domestic violence, lower frequency of husbands coming home, as well as less stable marriages. For low feeling of security, additional risk factors were the existence of sexual harassment after husbands left and domestic violence, longer left-behind duration, less stable marriage, as well as worse relationships with children. Our findings presented an overall picture of the psychological well-being of RLW in Northwest China and shed light on the factors associated with their compromised psychological well-being, providing valuable insights for implementing measures aimed at improving their overall living conditions and psychological well-being of RLW in Northwest China.

The results of the current study show that the psychological well-being status of RLW in Northwest China is not optimistic, with over 1/3 RLW having symptoms of depression (35.7%) and anxiety (37.6%). Poorer psychological well-being among RLW has become a consensus (1, 13, 19), given the multiple responsibilities that they have to undertake after their husbands left. Left-behind status has also been proven to be a risk factor for anxiety and depression among RLW (20). Previous studies have indicated that RLW showed higher scores in Symptom Checklist-90 (SCL-90) than China’s national norm in Jiangsu province (12, 19) and Mianyang city, Sichuan province (21). Some studies compared Chinese rural women with left-behind status to those who were not left-behind status and found that RLW exhibit higher depression levels (1, 2, 13). Jin et al. (1) reported that the prevalence of depressive symptoms was 46.69% for the left-behind women, which is significantly higher than 18.84% for the non-left-behind women. Yi et al. (2) also found higher depression and stress levels in left-behind wives than those who are non-left-behind wives, even though they did not report the exact prevalences. Although we did not compare RLW with non-left-behind women, the prevalence of depression and anxiety symptoms in the current sample were higher than the overall prevalence in the rural population in China (22, 23). For instance, Liang et al. reported that the overall prevalence of depression for rural population in China was 20.09%, regardless of men and women (22). A recent study also documented the overall prevalence of anxiety for rural women was 6.62% (23). Although the comparison was indirect, the prevalence of depression and anxiety among RLW in Northwest China found in the current sample were 35.7 and 37.6%, respectively, which were significantly higher than those in the general population and comparable to the prevalence of depression among left-behind women in other parts of China [e.g., Ma’anshan city (1) and Liuyang city (2)]. Additionally, as we mentioned above, the low feeling of security of RLW is one of the “three big mountains” they bear. In fact, our study revealed that the feeling of security score among RLW was also lower than score in general population in China reported in another study (17), even though the comparison was indirect. Another study indicated that 61.7% RLW in Mianyang city reported feeling of insecurity (21). Despite there are few studies on the feeling of insecurity among RLW, current findings, in accordance with limited previous studies, also reflect that RLW in Northwest China also face high levels of insecurity. Therefore, the general psychological well-being of RLW was not promising in Northwest China, and urgent attention should be given.

We further explored the associated factors of psychological well-being among RLW so as to deepen our understanding. The labor intensity and physical health conditions consistently predicting higher levels of depression and anxiety, and lower feelings of security. These associations have also been demonstrated in previous studies (19, 24). Too heavy labor directly impairs RLW’s quality of life in all aspects (19). Long-term heavy labor not only makes the body exhausted but also greatly reduces disposable time of themselves, resulting in the inability to release the accumulated psychological pressure. Meanwhile, physical health was closely associated with psychological well-being. In the current study, 47.6% of RLW reported they have common or serious diseases and suffering from physical diseases makes their living conditions even more severe as they perform heavy labor (24). Moreover, diseases also aggravate the economic pressure on the family, leading to greater psychological pressure for RLW. Additionally, specific factors were associated with depression, anxiety, and feeling of security. Due to the long separation from their husbands, RLW not only feel lonely since they lack the company of their spouses but also have various worries, such as doubting their husbands’ fidelity, which could also influence the psychological well-being of RLW. Our results found that marital satisfaction was associated with depression (the more marital satisfaction, the lower depression level), while marital stability was associated with anxiety (the more marital stability, the lower anxiety level) and feelings of security (the more marital stability, the higher feelings of security scores). Meanwhile, relationships with children and sexual harassment were related to depression and feelings of security. For RLW, children are an important source of social support, especially in the case of separation from the husband. Poor relationships with children make it difficult for RLW to receive emotional support, resulting in depressed mood and feelings of insecurity. Our results are consistent with previous studies that the lower the level of social support in RLW, the worse the psychological well-being would be (2). Some RLW also suffer from sexual harassment after their husbands left, which could seriously affect the psychological well-being of RLW, resulting in negative moods and feelings of insecurity. In this study, 5.6% RLW reported that they had been sexually harassed after husbands left, which should be taken seriously although the proportion is small. The present study shows domestic violence as another risk factor of poor psychological health, which has also been demonstrated in the previous study (1). In the present study, over 1/3 of RLW reported domestic violence. Domestic violence has been proven to cause high risk of mental disorders (25), and almost 15–71% of women worldwide experienced violence over their lifetime (26). The psychological trauma borne through domestic violence will seriously affect their psychological well-being. The frequency of the husband coming home was shown to be associated with anxiety, suggesting the more frequent the husband coming home, the lower anxiety level the RLW experienced. Consistent with Miao et al. (24), the husband works outside the home for a long time, causing RLW to have many worries and doubts, thereby leading to mental pressure and psychological problems. Meanwhile, the low frequency of husband coming home makes it difficult for RLW to get support and share from the husband both psychologically and in terms of labor pressure. Furthermore, we found that left-behind duration was an additional risk factor for feelings of insecurity. Zhang et al. (27) research on the RLW found that compared with RLW who stayed behind for less than 2 years, RLW who stayed for more than 2 years had poorer psychological well-being and overall survival status. Staying behind for a long time might increase the sense of uncertainty about marriage and the future, and thus decrease their feeling of security.

We did not find the associations of the psychological well-being with age, education level, the number of children, education pressure of children, marriage duration, and relationships with parents-in-law, even though they exhibited significant differences in one or more psychological indexes when using chi-square tests. Nevertheless, we should also pay attention to the influence of these variables since they have been found to be related psychological factors in other studies. For example, previous studies have shown that psychological well-being was associated with age (1, 27–29), education level (19, 24, 27), and relationships with parents-in-law (19, 24). Contrary to what we expected, we found no effect of religious beliefs on depression, anxiety, as well as feelings of security. As we stated before, previous studies have shown that religious beliefs could improve the ability of one to cope with stress and hardship, depression, and anxiety (14), but the current study indicated that “having religious beliefs” do not have a protective effect on psychological problems in Chinese RLW as it does in Western countries (30). This finding was consistent with the previous study in Ma’anshan city (1) indicating that religious belief was not a buffer of mental disorder in Chinese RLW as it in the west (14). On the contrary, higher risk for depression in RLW with religious beliefs was found compared with those without religious beliefs (1). It is noted that, although we did not find the significant association between religious belief and psychological well-being indexes, but further exploratory analysis revealed Tibetan Buddhism belief was a protective factor for depression, rather than anxiety, which is a different finding from this study, and was supported by previous study. As Sun and Qi demonstrated, notions of Tibetan Buddhism, as well as religious practices, could help with generating resilience at the level of response behavior (31). It is unclear why Islam was not a protective factor, and other religious beliefs even increase the burden of mental health. But Koenig pointed out that it is difficult to determine whether religious beliefs are a resource or a liability for people (32). Jin et al. also indicated that the RLW may try to find spiritual reposing in order to avoid the reality after frustration, which may be the reason why religious beliefs did not show protective effect on psychological well-being for RLW (1).

Overall, the present study provides an overall picture of psychological well-being of RLW in Northwest China, and its risk and protective factors that associated with psychological well-being. As an important indicator to measure the quality of life, psychological well-being should be given special attention. In the present study, although 1/3 of the RLW had depression and anxiety status, the proportion of severe was relatively small, suggesting the implementation of prompt and effective psychological interventions can have a positive and beneficial impact. For example, psychological health could be improved by creating safer rural communities, supporting the productive labor of RLW, enhancing rural medical services, and increasing access to psychological resources such as social support networks and mental health education. It is worth noting that our regression model for depression, anxiety, and feeling of security only account for 26.4, 33.2, and 18.9% of the variances, respectively, which means there are still several associated factors that have not been accounted for (e.g., coping strategies, social support, etc.) and should be further explored in the future.

There are inevitably some limitations in this study. Firstly, the current study is a cross-sectional design, which limits the determination of causality. Future studies would especially benefit from longitudinal design to determine causal relationships. Secondly, psychological well-being indexes were assessed by using self-rated measures, which inevitably introduces potential bias from recall errors or social desirability. Future research may benefit from a design combining both self-report and observer ratings. Thirdly, the current study only enrolled rural left-behind women, which makes difficult to compare the differences between rural women who are left-behind and those who are not left-behind, which limits our conclusion. Lastly, psychological well-being covers a wide range, we only include three representative variables (depression, anxiety, and feeling of security). More comprehensive measurement tools and even clinical interviews should be used for future studies. Nonetheless, the current study provided an overall picture of the psychological well-being of RLW in Northwest China. It also highlights the factors that contribute to the psychological well-being of RLW, which helps implement interventions and strategies aimed at improving their psychological well-being.

## Conclusion

In conclusion, the overall psychological well-being of RLW in Northwest China was not promising. The present study demonstrated that the detection rates of depression and anxiety status in RLW were 35.7 and 37.6%, respectively, and the feeling of security level in RLW was relatively low. The labor intensity, physical health conditions, marital satisfaction and stability, relationships with children, frequency of husband coming home, left-behind duration, domestic violence, and sexual harassment after husbands left were risk factors for the psychological well-being of RLW in Northwest China. Our findings may have implications for offering RLW accessible and targeted support and interventions they need to cope with their challenges, and thus improve their psychological well-being and living conditions.

## Data Availability

The datasets presented in this article are not readily due to privacy or ethical restrictions. Requests to access the datasets should be directed to the corresponding author.
