# Supplementary material for: Psychological well-being of rural left-behind women in Northwest China and its associated factors: a regional, population-based study
Source: Front Public Health. 2024 Dec 19;12:1395996. doi: 10.3389/fpubh.2024.1395996 (PMC11693742; doi:10.3389/fpubh.2024.1395996)
Supplement: Supplementary file 1 [file Table_1.DOCX]

SUPPLEMENTARY MATERIALS

**Psychological well-being of rural left-behind women in northwest China and its associated factors: A regional, population-based study**

**Table S1** Psychological well-being among different regions and ethnic groups

| **Indexes** | **Gansu**  **(G1; n=142)** | **Shanxi**  **(G2; n=171)** | **Ningxia**  **(G3; n=135)** | **Qinghai**  **(G4; n=150)** | **Xinjiang**  **(G5; n=99)** | ***F*** | ***p*** | **Post-hoc test**  **(Bonferroni)** |
| --- | --- | --- | --- | --- | --- | --- | --- | --- |
| **SDS** | 45.22 (12.11) | 48.98 (13.09) | 47.53 (12.93) | 40.80 (11.20) | 43.61 (10.78) | 10.654 | < .001 | G1&G2&G3>G4; G2>G5 |
| **SAS** | 49.48 (14.38) | 46.18 (11.79) | 47.40 (14.03) | 43.33 (11.66) | 44.73 (11.56) | 4.867 | < .001 | G1>G4&G5 |
| **SQ** | 50.01 (10.52) | 49.41 (12.98) | 48.44 (10.65) | 51.03 (10.50) | 52.72 (11.42) | 2.460 | 0.044 | G3<G5 |
|  | **Han**  **(G6; n=424)** | **Hui**  **(G7; n=112)** | **Uighur**  **(G8; n=10)** | **Tibetan**  **(G9; n=58)** | **Other**  **(G10; n=93)** | ***F*** | ***p*** | **Post-hoc test**  **(Bonferroni)** |
| **SDS** | 46.23 (12.95) | 45.28 (12.63) | 44.13 (8.08) | 39.14 (9.77) | 45.88 (11.05) | 4.253 | 0.002 | G6&G7&G10>G9 |
| **SAS** | 46.49 (12.87) | 45.16 (13.70) | 49.10 (11.03) | 42.20 (11.41) | 48.86 (12.56) | 2.765 | 0.027 | G10>G9 |
| **SQ** | 50.16 (11.77) | 50.03 (10.88) | 50.59 (11.07) | 51.69 (10.99) | 49.31 (10.43) | 0.399 | 0.809 | — |
| ***Abbreviations***: SDS, self-rating depression scale; SAS, self-rating anxiety scale; SQ, security questionnaire. | | | | | | | | |

**Table S2** Psychological well-being among different religious beliefs

| **Indexes** | **Non-religious beliefs**  **(G1; n=352)** | **Islam**  **(G2; n=204)** | **Tibetan Buddhism**  **(G3; n=112)** | **Other religious beliefs**  **(G4; n=29)** | ***F*** | ***p*** | **Post-hoc test**  **(Bonferroni)** |
| --- | --- | --- | --- | --- | --- | --- | --- |
| **SDS** | 45.39 (13.00) | 45.84 (11.83) | 42.97 (11.86) | 51.98 (10.73) | 4.234 | 0.006 | G3<G2<G4; G1<G4 |
| **SAS** | 45.86 (12.57) | 47.18 (13.27) | 45.45 (13.87) | 48.01 (10.00) | 0.781 | 0.505 | — |
| **SQ** | 50.61 (11.55) | 49.53 (10.74) | 51.78 (10.83) | 42.94 (12.95) | 5.136 | 0.002 | G1&G3>G4 |
